# Gene Expression Profiling as a Tool to Investigate the Molecular Machinery Activated during Hippocampal Neurodegeneration Induced by Trimethyltin (TMT) Administration

**DOI:** 10.3390/ijms140816817

**Published:** 2013-08-15

**Authors:** Wanda Lattanzi, Valentina Corvino, Valentina Di Maria, Fabrizio Michetti, Maria Concetta Geloso

**Affiliations:** Institute of Anatomy and Cell Biology, Università Cattolica del Sacro Cuore, Largo F. Vito 1, Rome 00168, Italy; E-Mails: wanda.lattanzi@rm.unicatt.it (W.L.); valentina.corvino@rm.unicatt.it (V.C.); valentinadimaria1@virgilio.it (V.D.M.)

**Keywords:** trimethyltin, neurodegeneration, gene expression profiling, hippocampus, animal models, cell culture

## Abstract

Trimethyltin (TMT) is an organotin compound exhibiting neurotoxicant effects selectively localized in the limbic system and especially marked in the hippocampus, in both experimental animal models and accidentally exposed humans. TMT administration causes selective neuronal death involving either the granular neurons of the dentate gyrus or the pyramidal cells of the Cornu Ammonis, with a different pattern of localization depending on the different species studied or the dosage schedule. TMT is broadly used to realize experimental models of hippocampal neurodegeneration associated with cognitive impairment and temporal lobe epilepsy, though the molecular mechanisms underlying the associated selective neuronal death are still not conclusively clarified. Experimental evidence indicates that TMT-induced neurodegeneration is a complex event involving different pathogenetic mechanisms, probably acting differently in animal and cell models, which include neuroinflammation, intracellular calcium overload, and oxidative stress. Microarray-based, genome-wide expression analysis has been used to investigate the molecular scenario occurring in the TMT-injured brain in different *in vivo* and *in vitro* models, producing an overwhelming amount of data. The aim of this review is to discuss and rationalize the state-of-the-art on TMT-associated genome wide expression profiles in order to identify comparable and reproducible data that may allow focusing on significantly involved pathways.

## 1. Introduction

The trisubstituted organotin compound trimethyltin chloride (TMT) is a potent neurotoxicant that causes, in mammalian brain, selective neuronal death specifically localized in the limbic system and, in particular, in the hippocampus [1,2]. Being widely used in both industry and agriculture as a constituent in fungicides and in plastic production [3], some cases of occupational or accidental human exposure to TMT have been described [2,4–6]. Exposed individuals exhibit neuropathologic and behavioural features similar to those that are elicited in experimental animal models of TMT intoxication, which thus offer a promising model of neurodegeneration, also susceptible to translational exploitations.

In rodents, TMT-administration induces behavioural alterations (hyperactivity and aggression), cognitive deficits (memory loss and learning impairment) and seizures [7,8], as a consequence of selective loss of specific hippocampal neuronal subpopulations, accompanied by reactive astrogliosis, microglial activation and enhanced neurogenesis. Consequently, it offers a valuable tool to study neuro-glial interactions associated with neuronal death processes and cellular and molecular events related to injury-induced neurogenesis [9–12]. Interestingly, TMT-induced hippocampal injury is not accompanied by blood–brain barrier (BBB) disruption [13–15], thus offering an instrument to study pathogenic events occurring before BBB impairment, including early resident microglial responses to neuronal damage [16].

The molecular mechanisms by which TMT induces selective neuronal death are still not conclusively clarified: different pathogenetic pathways, probably acting differently in *in vivo* and *in vitro* models, seem to be involved, including neuroinflammation, intracellular calcium overload, and oxidative stress [7,17,18].

At the cellular level, mitochondrial dysfunction has been proposed as a possible causative factor of cell death [19,20], through the involvement of a mitochondrial membrane bound protein termed stannin selectively expressed by TMT-sensitive cells [21].

Overall, the complete molecular scenario involved in TMT-induced neurodegeneration is still far from being clearly identified, though interesting insights have been obtained through genome-wide technologies, such as microarray analysis, aimed at global comparative gene expression profiling. Microarray analysis is a powerful investigative tool in molecular biology, as it allows the simultaneous expression profiling of the entire genome and has thus become a key technology in toxicology research [22]. To date, microarray technology has been employed to unravel the molecular mechanisms acting during TMT intoxication in distinct studies performed in the different models of TMT-induced neuronal death: the *in vivo* mouse and rat models and an *in vitro* cell culture model [10,15,23–27]. The aim of this review is to discuss and rationalize the state-of-the-art on gene expression profiling data regarding the TMT intoxication model, in order to identify comparable features that may allow focusing on significantly involved pathways.

## 2. Different Models Used to Investigate TMT-Induced Gene Expression Profiling

The neuropathological features of TMT-induced hippocampal damage differ among rodent species, depending on various parameters such as strain, age, dose, route of administration, essentially as a consequence of differences in metabolism and kinetics of the toxicant [1,28–30].

Therefore TMT-treatment offers at least two *in vivo* animal models for the study of different aspects of injury induced-neuronal death: a model of acute dentate granule cell apoptosis in mice, occurring within 48 h from TMT-administration, and a model of progressive CA1/CA3 pyramidal cell death in rats, developing over three weeks. The mouse model of TMT-induced selective granular cell apoptosis is mainly used to investigate early molecular events involved in neuronal death. Conversely, in the rat model, the intoxication is characterised by a subacute course; thus it is widely considered a model of chronic neurodegeneration [18,31,32] and, according to some authors, it had been even regarded as a model resembling some features of Alzheimer’s disease [33–36]. Since cell cultures have been usefully used to delineate selective features of TMT-induced neuronal death and to depict the specific role of glial cells in TMT-induced intoxication [37–46], gene expression profiling data have also been obtained in a homogeneous cellular model [27].

### 2.1. The Mouse Model

TMT-induced lesions in mice selectively affect dentate gyrus (DG) granule cells [1], with a different level of vulnerability depending on strain [7,47,48]. The involvement of the olfactory bulb and of the anterior olfactory nucleus has also been reported [49]. Neuronal death is induced by apoptosis, as demonstrated by chromatin condensation, DNA fragmentation and activated caspase-3 in degenerating granular cells [50,51].

Gene expression profiling data from murine hippocampus following TMT intoxication indicate the activation of molecular pathways involved in calcium homeostasis, inflammation, neurodegeneration, neurogenesis and apoptosis. In particular, relevant hints towards the clarification of the molecular scenario involved in TMT-induced selective brain damage could be gained from the original study by Lefebvre d’Hellencourt and Harry in the comparative expression profiling between the DG granular cells, selectively affected by the toxicant, and the essentially unaffected Cornus Ammonis (CA) pyramidal neurons, microdissected from the murine hippocampus (see Table 1). These authors observed, as early as 6 h post-TMT treatment in the DG of young mice (P21), a significantly increased expression of genes involved in neuronal differentiation and astrocyte activity (e.g., *PEA-15* and *NeuroD1*), in inflammatory response (*T-cell antigen receptor* and *CD3 antigen zeta*, among others), in cell adhesion (*cadherin 5*, *CD14*) and in apoptosis (*DNA-damage inducible transcript 3*, *DDIT3*) [24]. Conversely, they found only a slight expression modulation of the same genes in the CA at the same time point. At 18 h post-treatment, a significant overexpression of genes involved in neuronal survival (*ATF3* and *ATF4*), cell cycle activation (*cyclin D2*, *CDK5* and *CDK7*), migration (*MIP1 alpha* and *beta*, for instance) and differentiation (such as *HSP70-5*, *inhibin beta-A* and *hairless*) occurred in the DG [24]. At 18 h, some of these transcripts (namely *ATF3*, *ATF4* and *DDIT3*) displayed an even higher up-regulation in the CA region. The authors interpreted these data as specifically indicative of a cell survival response due to the presence of spared neurons in the DG (around 50%–60%) and in the whole CA region [24].

These findings would suggest that the acute response occurring in the target region of TMT intoxication (the DG) is represented by an early inflammatory response, followed by a stress-induced attempt of rescue, sustained by cell cycle activation, neuronal differentiation and glial activation. Overall, these observations allow hypothesizing that TMT-induced brain injury in the young murine model evokes a neurogenic stimulus.

Notably, most gene expression changes in this experimental condition were negative (*i.e.*, expression inhibition). Downregulated genes included transcription factors, structural proteins, calcium regulators and signaling molecules, which did not display a significant differential expression across the two regions, indicating some kind of generalized molecular response across the whole hippocampus [24]. Interestingly, a higher extent of downregulation of genes involved in calcium homeostasis (including *calbindin-28k*, *calmodulin* and *calcineurin*) was observed in the DG. This functional group of genes actually displayed higher basal expression in the DG compared to the CA region, representing a possible hallmark in the DG molecular profile.

The hippocampal molecular profile of TMT intoxication has been also assessed at later time points in other studies (see Table 1).

Funk and colleagues used the TMT-treated mouse to assess the neuroprotective role exerted by physical exercise on a brain injury model [15] (see Table 1). In this study, the gene expression profiling of the whole hippocampus 24 h post-TMT treatment allowed evidencing a prominent role of caspase-dependent apoptosis, in line with previous morphological evidence [50,51]. In particular, a significantly increased expression was observed for both *caspase 3* and *caspase 8*, along with the *nuclear factor of kappa light polypeptide gene enhancer in B-cells 1* (*NFkB 1a*), which blocks the nuclear translocation and phosphorylation of NFkB [52]. NFkB is known to be involved in the TMT-induced neurodegenerative events, participating in both neuronal survival related processes [23,53] and in neuronal damage [18,54,55]. In this case, it could be activated as a downstream messenger of the *tumor necrosis factor alpha* (*TNFalpha*)-dependent cascade driving the apoptotic process [56]. A slight but significant upregulation was also observed for the *tumor necrosis factor receptor superfamily member 25* (*Tnfrsf25*), an agonist for the TNF receptor [15], thus confirming previous experimental data from *in vivo* and *in vitro* studies [37,44,45,57], and further supporting the specific role for TNF receptor signaling in TMT-mediated neuroinflammatory processes [45]. Interestingly, both *NFkB 1* and *TNFalpha* are strongly upregulated in the hippocampus of patients affected by temporal lobe epilepsy associated with hippocampal sclerosis, supporting the role of neuroinflammation in human epilepsy [58] and the translational value of the molecular mechanisms activated by TMT in rodent hippocampus. Notably, the expression modulation induced by the TMT treatment in genes involved in cell death pathways was attenuated in mice with prior exercise, consistent with the postulated neuroprotective role [15]. As a whole, most differentially expressed genes that resulted during this study were annotated in inflammation and cell death functional categories. In particular, the interleukin-6 (IL-6)-related pathway was strongly activated by TMT, even in animals that performed exercise. The authors also found the upregulation of genes involved in cell survival and apoptosis inhibition (*Hras1*, *Fos oncogene*, the *signal transducer and activator of transcription-3* (*Stat3*) gene) [59]. Altogether, these data should point towards a cell survival strategy driven by pro-inflammatory signals, put in place within the murine hippocampus 24 h after TMT treatment [60,61]. These events could depict the molecular profile of glial activation occurring within the acute effects of TMT intoxication in the mouse model [8].

The same authors recently examined the modulation of gene expression profile of mice subgranular zone (SGZ) during TMT intoxication (see Table 1). They focused their attention, in particular, on the differential expression of *IL-1alpha* and *IL-6* genes, as a function of age during TMT intoxication. The transcript expression pattern confirmed that the IL-6-mediated pro-inflammatory signaling is involved in the TMT neurotoxicant effects displayed in the SGZ of adult (one year old) mice [10]. Conversely, they demonstrated that an *interleukin 1* (*IL-1*)-related immune activation occurs in the SGZ of adolescent mice (P21), 48 h after TMT administration. The two alternative pathways may indeed reflect the age-related differences in proliferative response of neural progenitors upon the neurotoxicant administration. The original molecular scenario delineated in this study proposes an IL-1alpha-related signaling, involving *NFkB 1* among other genes overexpressed in adolescent compared to adult mice, as a putative alternative neurogenesis pathway [10].

In this respect, the study performed by Kassed and colleagues [23] (see Table 1), focusing on the pro-survival role of NFkB during the late phases of TMT-induced neurodegeneration, appears to be noteworthy. Gene expression profiling was studied by comparing TMT-treated wild type (wt) and genetically manipulated mice lacking the p50 subunit of NFkB protein complex (p50-null mice) with saline-injected controls at post-treatment day 7.

Genes related to immune function (e.g., *Complement C1qB*, *chemokine CXC ligand 13*), potential neuroprotective genes as heat shock proteins (e.g., *Hsp70* and *HRP12*), lysosomal enzymes (e.g., *beta-glicuronidase*, *cathepsin C* and *D*), genes related to cell surface proteins (e.g., *CD45* and *CD53*), and the calcium/calmodulin dependent protein kinase II were upregulated in wt TMT-treated mice, but not in p50-null mice. Overall, this profile further supports the role of NFkB in their regulation. Interestingly, the array results showed that the up-regulation of genes related to survival and brain repair is still upregulated in the late phases of TMT-induced granule cell death in mice.

**Figure 1:**
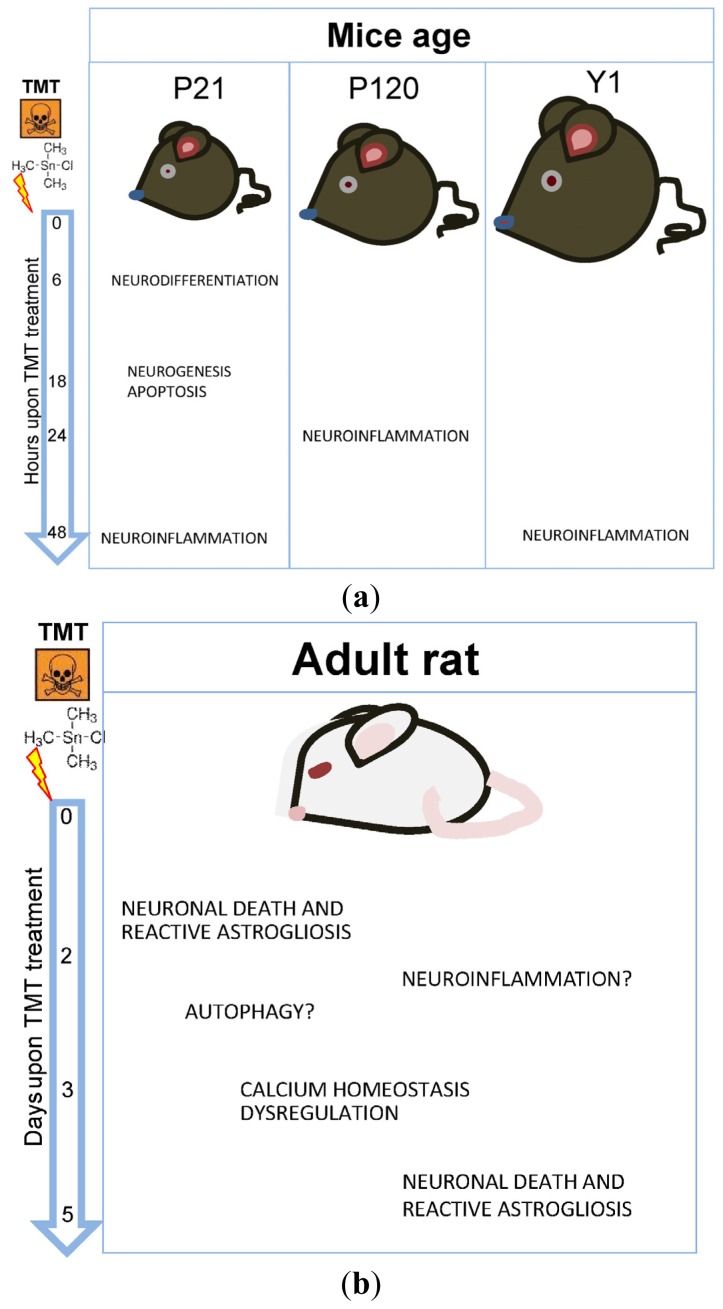

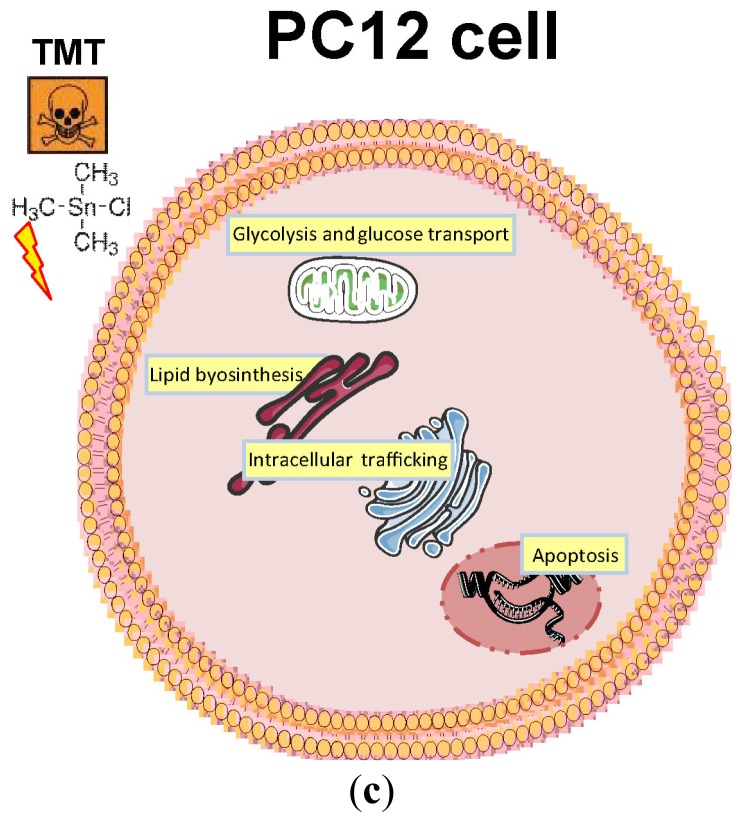
A schematic representation of TMT-induced gene activation in (**a**) mice; (**b**) rats; and (**c**) PC12 cells. (**a**) Results obtained in mice indicate the significant and early involvement of genes related to apoptosis, neuroinflammation and neurogenesis; (**b**) The studies performed in the rat model reflect an involvement of calcium homeostasis dysregulation and mitochondrial damage. The contribution of the authophagic pathway may be hypothesized, but genes related to neuroinflammation are not unambiguously modulated in the early phases of TMT-induced neurodegeneration; and (**c**) the study performed in PC12 cells evidences the activation of oxidative metabolism of glucose, cholesterol and fatty acids, along with the early activation of genes involved in apoptosis and stress-related pathways.

Taken together, the results obtained in gene expression profiling studies performed in mice appear to homogeneously support current hypotheses on the main pathogenic mechanisms involved in TMT-induced acute granule cell death: alteration of calcium homeostasis, apoptosis and neuroinflammation. Genes associated with the tissue reaction (glial activation and neurogenesis) are also clearly activated (Figure 1a). A central role of neuron/microglial interactions emerges in driving neuroinflammatory signals leading to TMT-induced neuronal death. In addition, data from SGZ studies further support the role exerted by microglial cells on injury-induced neurogenesis [10,62,63].

It is noteworthy in this regard that the stimulation of repair mechanisms has been described in both early and late phases of TMT-induced granule cell death [23,24]. This finding suggests that the mouse model could be suitable to study the molecular mechanisms involved in brain-injury associated with neuroreparative processes, occurring in different temporal frames.

### 2.2. The Rat Model

After TMT administration, rats develop extensive lesions in the CA, typically localized in the CA3/Hilus and also involving CA1, while granular neurons in the DG are generally spared [1,28]. Neuronal death shows a delayed onset (two days after treatment) and progressively worsens: it develops over three weeks [7,8,17], probably on account of the high affinity of rat hemoglobin for TMT [17,29], involving CA3 earlier and more severely than CA1 [12,64].

In addition, it occurs in pyramidal cells, with selective sparing of Parvalbumin and Calretinin interneurons, both in adult and in developing rats [65–67]. Also, in the rat model, early astroglial [28,68,69] and microglial activation [58,70,71] are always associated to TMT-induced neuronal death. Thus, the early molecular events, playing a causative role in pyramidal neuronal death, may be different in the two rodent species. The two studies performed on gene expression profiling on the rat model [25,26] evaluated two time points in the early phases of TMT-induced neurodegeneration (see Table 1). The authors explored earlier time points, specifically two to three days after treatment [25,26], corresponding to the earlier evidence of neuronal death [28]. They also analyzed the molecular profile five days after intoxication, when hippocampal neuronal death, reactive astrocytosis and activation of microglia are well established [32,72]. Morita and colleagues did not show microarray results at the two to three day time point, but only the fold changes of differential gene expression confirmed by quantitative reverse transcriptase polymerase chain reaction (qRT-PCR). They validated only those genes whose expression changed after TMT and FK-506 coadministration, as their aim was to evaluate the neuroprotective role of this drug against TMT-induced neurodegeneration [25].

The study by Little and colleagues [26] pointed out interesting data related to the first time point examined. The authors stressed the activation of genes involved in astroglial activation, such as those related to the glial proteins GFAP and 14-3-3, which, along with the down regulation of *SNAP25*, may suggest loss of synapses. The decreased expression of cell structure genes, such as *tubulin* and *MAP1B*, essentially reflects the phenomena of neuronal loss and astrocytic reaction, occurring at hippocampal level.

Moreover, this study confirmed at the transcriptional level the role exerted by mitochondrial damage in TMT-induced neuronal death, as already indicated by previous data [20,46].

Intriguingly, a relevant group of genes modulated in the early phases of neurodegeneration [26]—in particular, the lysosomal membrane protein *LAMP1*, *Presenilin* (*PS*) *1*, *14-3-3*, *RAB8B* and the *heat shock protein of 90 kDa chaperone complex (HSP90)*—are also involved in the autophagic pathway [72–76], which has recently been suggested to play a role in TMT-intoxication [77]. It is known that autophagy is involved in the degradation of protein aggregates and damaged organelles, among which damaged mitochondria and alterations of autophagy can even lead to neuronal death [78].

The modulation of ubiquitin proteasome related genes, as well as *clathrin*, both involved in exosomes protein selection machinery [79], might also suggest a modulation of exosome signaling in the injured hippocampus, giving rise to new cues for further investigation.

In addition, the involvement of LAMP1 and both PS1 and PS2 [26] in intracellular Ca^2+^ homeostasis [80,81] appears to be in line with the hypothesis that TMT-induced neuronal damage could be largely dependent on calcium homeostasis dysregulation [82–85].

Data from Morita and colleagues [25], though not discussed by the authors and reported only at the later time point examined, support this observation. In fact, a modulation of other genes related to calcium signaling, such as *preprocathepsin* and *cathepsin D* (upregulated) and the *vesicle associated calmodulin binding protein*, and *calcineurin*, downstream target of the Ca^2+^-calmodulin complex (downregulated) [86], is also reported. A downregulation of *Cadherin EGF LAG seven path G-type receptor (Flamingo)* gene, a type of non-classical cadherin molecule [87], is also observed in this study.

*NFkB* gene appeared to be downregulated in the early phases of TMT-induced neuronal damage in the study by Little and colleagues [26], although the overexpression of the pro survival NFkB p50 protein subunit was previously demonstrated by immunohistochemistry in the rat hippocampus two to seven days after TMT-treatment [88]. The authors associated this event primarily to the absence of early activations of inflammatory mediators, concluding that, in the rat model, neuroinflammation does not play a causative role in initiating neuronal death.

The results by Little and colleagues are not in agreement with those by Morita and colleagues, who found a significant upregulation of some genes associated with the inflammatory response, including *Matrix Gla protein* gene, *lysozyme* and *osteopontin*, though failing in detecting changes in the expression of inflammatory markers (e.g., *IL-1alpha* and *TNFalpha*). In particular, osteopontin acts in many tissues as a critical cytokine for macrophage recruitment and activation during cell-mediated immunity [89]. Interestingly, osteopontin has also been found to be upregulated in degenerating neurons in other models of epilepsy [90]. This study also supports the role of oxidative stress-related cell death, through the upregulation of *anti-glutathione S transferase* [25], both two and five days after TMT-treatment. The expression of both inflammatory and oxidative stress-related genes is further increased five days after TMT-treatment and, interestingly, it is reversed by FK-506 administration [25].

Although the results on neuroinflammation may appear conflicting, it is interesting to note that, in the rat model of TMT-induced neurodegeneration, the early expression of neuroinflammatory mediators has been described both *in vitro* [91] and *in vivo* [92]. In addition, the astroglial expression of IL-1alpha and IL-1beta [93], Cyclooxygenase-2 [94] and proteinase activated receptors 1 and 2 [69] have been reported at later time points (14 days post treatment). Since in the mouse model the expression of neuroinflammatory mediators was essentially described to occur in microglia [95], this observation further evidences a difference in the cellular source, which may contribute to the species’ difference.

In addition, we might speculate that neuroinflammation, which plays a causative role in granular neurons death in the mouse model of TMT-induced neurodegeneration, exhibits a different temporal profile in the rat model. Further studies would be needed to better clarify the specific role of neuroinflammation in the worsening of the ongoing hippocampal degeneration in rats.

Both Little’s and Morita’s studies report an upregulation of genes related to the insulin growth factor (IGF) pathway (respectively, *IGF receptor 2* and *insulin-like growth factor binding protein-2*) starting at the earliest time points of TMT-induced hippocampal damage and detectable also five days later [25]. Blood IGF1 and IGF2 levels exert trophic effects on neurogenesis and neuronal survival [96], as already suggested in the mouse model [97]; it has also been recently shown that cultured human microglia are significant sources of IGF1 and IGF2 that can be modulated by inflammatory cytokines [98].

Data regarding the second time point explored in the two above mentioned studies [25,26] (5 days after TMT-administration) essentially reflect the events characterizing the neuropathological features of TMT-induced lesion at this time point post-treatment (neuronal loss and glial activation). The authors describe an increased expression of genes considered markers for activation of microglia (*CCL2*, *MHC-I* and *MHCII*), astroglia (*GFAP*, *vimentin*, *glutamine synthetase* and *peripheral benzodiazepine receptor*), and genes related to neurodegeneration such as *PS2*, *β-catenin*, and *Tau*. In line with these findings, Morita *et al.* [25] refers to an increase of *S100* related protein mRNA, possibly linked to the developing astroglial reaction, and a downregulation of genes related to neurotransmission and synaptic function, such as *Synaptotagmin XI* [99], *Synaphin 2* [100] and *synaptic cell adhesion molecule 1* [101]. These data, along with the expression changes of the GABA transporter alanine-sensitive protein [102] and of the *neuropeptide Y* gene [25], may reflect the loss of synapses and neurotransmitter alterations related to seizures, neuronal loss and related plasticity changes [17].

Taken together, the information coming from gene expression studies performed in the rat model reflects a more heterogeneous scenario, when compared with those derived from mice. An involvement of calcium homeostasis dysregulation, as well as mithocondrial damage, are clearly indicated (Figure 1b). The contribute of the authophagic pathway may be hypothesized, while genes related to neuroinflammation appear not to be unambiguously modulated in the early phases of TMT-induced neurodegeneration in rats, and a definite molecular pathway playing a causative role in pyramidal neurons death does not seem to emerge in the reported data.

This could be due to many factors. The processes underlying neurodegenerative phenomena occurring in mice and rats may reasonably be different, the first being an acute phenomenon, while neurodegeneration occurring in rats may be regarded as a chronic phenomenon. In addition, as the onset of neuronal death may delay two to four days after treatment [28], individual differences among treated rats are possible and a larger group of animals could be needed to achieve conclusive results.

Moreover, the examined specimens (whole hippocampi) are reflective of an heterogeneous cell population (neurons and glia), and slight differences in the expression of genes involved in different pathways, possibly leading to opposite effects (neuronal death *versus* neuroreparative processes), may be underestimated in these conditions.

In addition, differences in rat strains, in the dosage schedule and route of administration used in the different studies (see Table 1) should be taken into account and may explain some of the observed discrepancies.

### 2.3. The Cell Culture Model

Cell cultures offer a simplified model to study molecular mechanisms, as they allow examining a more homogeneous system, although they lack information deriving from cell-cell interaction and from microenvironment of the *in vivo* tissue. Thus, an *in vitro* model to study cell events leading to death upon TMT intoxication has been proposed using a homogenous cell line, the rat pheocromocytoma PC12 cells, commonly used for studying cell proliferation, neurotoxicity and cell death. The results obtained in this study demonstrated that sub-lethal concentrations of TMT induce adaptive mechanisms in the cell, through the activation of oxidative metabolism of glucose, cholesterol and fatty acids, along with the early activation of genes involved in apoptosis and stress-related pathways [27] (Figure 1c). Also, the intracellular trafficking was apparently activated while calcium transport was not evidently involved in this model, except for the upregulation of two genes (*calumenin* and *reticulocalbin 3*). A possible keystone to interpret the concurrent activation of metabolic and injury-related signaling is represented by a response to hypoxic stimulus. In fact, many genes in the gene list were targets of the transcription factor encoded by the hypoxia-inducible gene 1 (*Hif1α*), which was among the upregulated genes. HIF1α represents a key factor of hypoxia-related intracellular reactions, as it generates adaptive responses by inducing the expression of various target genes involved in proliferation, glucose metabolism and transport [103]. On the other hand, the same molecule may induce apoptosis through the activation of cytokines, such as *Bnip3* (also upregulated in PC12 cells upon TMT treatment), which cause mithocondrial dysfunction [20,27,103]. HIF1α has also been recently shown to modulate the proliferation/differentiation switch in neural stem and progenitor cells, in response to hypoxic and hypoxic-like noxae to the niche [104]. Interestingly, the metabolic profile observed in TMT-intoxicated cells indicates the overall activation of lipid biogenesis [27], which may reflect the known effects of organotin compounds on plasma membrane composition, plasticity and permeability [105–108]. Besides the pro-apoptotic effects, TMT seems to also induce autophagic cell death in PC12 cells, as suggested by the activation of selected genes (namely *Bnip3*, *Bnip3-like* and *adrenomedullin*) [27].

## 3. Concluding Remarks and Perspectives

Some general considerations may emerge when searching for common features in the molecular profiles associated to the different models studied, although it is difficult to compare or combine the results obtained from the microarray analysis, due to substantial differences in sample composition, tissue sources, and methodologies. As a rule, the consistency of microarray data largely depends on sample dimension and heterogeneity [109–112]. Such general observations perfectly apply to the case of microarray studies performed in the different TMT models that are characterized by relevant differences in the biological and technical features of the experimental protocols.

As expected, the different studies confirm the critical role played by mitochondrial dysfunction and disruption of Ca^2+^ homeostasis in the early phases of TMT-induced neuronal death [24,26,27].

Moreover, the different models share data on the modulation of genes involved in the autophagic pathway, such as those encoding lysosomal enzymes (e.g., beta-glicuronidase, cathepsin C and D, LAMP1) [25,26,53], *SNARE protein genes* [25] and *lipid biogenesis* [27], thus suggesting a possible role played by this pathway in TMT-induced neuronal death. Interestingly, it has been proposed that autophagy is involved in neurodegeneration through the inactivation of NFkB [113], which has been shown to be involved in TMT-induced neuronal death [18,24,54,55]. In conclusion, as TMT activates different pathogenic mechanisms leading to cell death, the approach based on gene profiling examination appears to be promising, since it provides a comprehensive snapshot of the molecular scenario. Nonetheless, further studies are needed to achieve a convincing functional validation of the specific role and possible intersection of the signaling cascades triggered by TMT administration and leading to selective neuronal death.

## Figures and Tables

**Table 1:** Overview of microarray-based genome-wide expression analysis in the *in vivo* trimethyltin (TMT)-induced models of neurodegeneration.

Model animal (No. of samples per experimental group)	Age at treatment	Rodent strain – Animal gender a	TMT dosage (administration route)	Hippocampal tissue specimen	Tested time points (post-TMT treatment)	Reference
Mouse (*n* = 3)	Adult	B6, 129 Nfkb1 ^tmlBal^ B6,129 2/J – F/M	p-50 null: 2.0 mg/kg; Non transgenic: 2.25 mg/kg	Whole hippocampus	7 days	[23]
Mouse (*n* = 10)	P21	CD-1 – M	3 mg/Kg (i.p.)	Microdissected DG and CA	6–18 h	[24]
Mouse (*n* = 3)	P120	CD-1 – M	2.4 mg/Kg (i.p.)	Whole hippocampus	24 h	[15]
Mouse (*n* = 3)	P21 1 year	CD-1 – M	2.3 mg/Kg (i.p.)	Microdissected SGZ	48 h	[10]
Rat (*n* = 3)	6 weeks	Sprague–Dawley – M	9 mg/Kg (oral)	Whole hippocampus	2–5 days	[25]
Rat (*n* = 3)	6 weeks	Long –Evans – F/M	8.0 mg/Kg (i.p.)	Whole hippocampus	3–5 days	[26]

a F: female; M: male; F/M: both sexes.
